# MicroRNA-143-3p, up-regulated in *H. pylori*-positive gastric cancer, suppresses tumor growth, migration and invasion by directly targeting AKT2

**DOI:** 10.18632/oncotarget.15646

**Published:** 2017-02-23

**Authors:** Fang Wang, Jiatao Liu, Yanfeng Zou, Yang Jiao, Yawei Huang, Lulu Fan, Xiaoqiu Li, Hanqing Yu, Chengqun He, Wei Wei, Hua Wang, Guoping Sun

**Affiliations:** ^1^ Department of Oncology, The First Affiliated Hospital of Anhui Medical University, Hefei 230022, Anhui, China; ^2^ Department of Pharmacy, The First Affiliated Hospital of Anhui Medical University, Hefei 230022, Anhui, China; ^3^ Department of Epidemiology and Biostatistics, School of Public Health, Anhui Medical University, Hefei 230032, Anhui, China; ^4^ Department of Gynaecology and Obstetrics, Anhui Provincial Hospital, Hefei 230001, Anhui, China; ^5^ Institute of Clinical Pharmacology, Anhui Medical University, Hefei 230032, Anhui, China; ^6^ Institute for Liver Diseases of Anhui Medical University, Hefei 230032, Anhui, China

**Keywords:** *Helicobacter pylori*, gastric cancer, microRNA-143-3p, AKT2, progression

## Abstract

Our previous studies have suggested a protective role for *H. pylori* infection in the prognosis of gastric cancer. Based on those findings, we hypothesized that *H. pylori*-positive and -negative gastric cancers may exhibit different growth patterns and pathobiological behaviors, indicating different mechanisms of cancer progression. By microarray analysis, we studied miRNAs expression profiles in 42 gastric cancer patients, comparing 21 *H. pylori*-positive and 21 *H. pylori*-negative groups. Luciferase reporter assay and western blot were used to examine the potential target genes of the interested miRNA. In the present study, 53 miRNAs were significantly differentially expressed in *H. pylori*-positive and -negative gastric cancer tissues. We investigated the expression and function of one candidate, miR-143-3p, which was the most significantly increased miRNA in *H. pylori*-positive gastric cancer tissues. We observed that miR-143-3p expression was significantly decreased in gastric cancer tissues and cells, which correlated with late stage and lymph node metastasis. Using gain- and loss-of-function experiments *in vitro*, we demonstrate that miR-143-3p negatively regulated cell growth, apoptosis, migration and invasion. We further characterized AKT2 as a novel direct target of miR-143-3p. Knockdown of AKT2 expression mimicked the effects of miR-143-3p restoration. In conclusion, our data suggest that miR-143-3p acts as a novel tumor suppressive miRNA by regulating tumor growth, migration and invasion through directly targeting AKT2 gene. Further investigation is warranted to characterize the mechanisms underlying gastric cancer progression and may eventually contribute to its therapy.

## INTRODUCTION

*Helicobacter pylori* (*H. pylori*) is a Gram-negative, microaerophilic bacterium that has been recognized as associated with gastric cancer by the World Health Organization [1]. Mounting evidence has shown linkage between *H. pylori* infection and the development of gastric cancer. Recently, studies have focused on the differences in survival of patients who are positive for *H. pylori* compared with those who are negative. Meimarakis et al. identified *H. pylori* as an independent, beneficial prognostic factor in a prospective cohort [2]. Our previous study confirmed *H. pylori* status as a favorable prognostic factor in a Chinese prospective cohort [3]. Furthermore, we performed a meta-analysis to derive a more precise estimate of the association, which suggested a protective role for *H. pylori* infection in the prognosis of gastric cancer [4]. Based on the evidence, we hypothesized that *H. pylori*-positive and -negative gastric cancers were quite different in growth patterns and pathobiological behavior, indicating different mechanisms of cancer progression. Further knowledge of the two different types of gastric cancer may help to elucidate the identification of novel therapeutic targets.

MicroRNAs (miRNAs) are a class of highly conserved short RNAs of approximately 17 to 24 nucleotides. miRNAs regulate diverse cellular processes by binding to their target messenger RNAs (mRNAs) within the 3′-untranslated region [5]. Numerous reports have demonstrated that miRNAs are involved in biological and pathological processes, such as tumorigenesis, cell proliferation, apoptosis, cell migration and metastasis [6–8]. Emerging evidence indicates that miRNA expressions are specifically modified in human gastric cancer versus non-cancerous adjacent tissue [9, 10]. Other concurrent studies have described global miRNA expression patterns in *H. pylori*-infected gastric mucosa [11]. To date, differentially expressed miRNAs between *H. pylori*-positive and *H. pylori*-negative gastric cancer remain to be explored. Given that misexpressed miRNAs are involved in the development and progression of *H. pylori*-associated gastric cancer, they could serve as therapeutic targets in gastric cancer.

The aim of this study was to elucidate miRNAs that are differentially expressed in *H. pylori*-positive and *H. pylori*-negative gastric cancer tissues using microarray technology. Next, we further studied the expression and functions of one hit, miR-143-3p, in gastric cancer. Luciferase reporter assays and western blots were used to examine potential target genes.

## RESULTS

### *H. pylori* infection remains a favorable prognostic factor for gastric cancer patients

In previous studies, we have identified *H. pylori* infection as an independent, favorable prognostic factor for patients who undergo curative resection of gastric cancer [3]. In this study, we updated the results of the previous study with respect to the follow-up through July 28, 2014. The mean overall survival was 73.0 months (95%CI: 70.2 to 75.7 months) in *H. pylori*-positive patients and 61.1 months (95%CI: 56.3 to 65.9 months) in those who were *H. pylori*-negative (*P*=0.037). The mean disease-free survival was 70.9 months (95%CI: 67.6 to 74.1 months) in *H. pylori*-positive patients and 58.8 months (95%CI: 53.4 to 64.1 months) in those who were *H. pylori*-negative (*P*=0.040). The Kaplan-Meier survival curves for overall survival and disease-free survival are shown in Supplementary Figure 1. The updated survival data were consistent with our previous findings.

### miRNA expression profiles in *H. pylori*-associated gastric cancer

**Figure 1:**
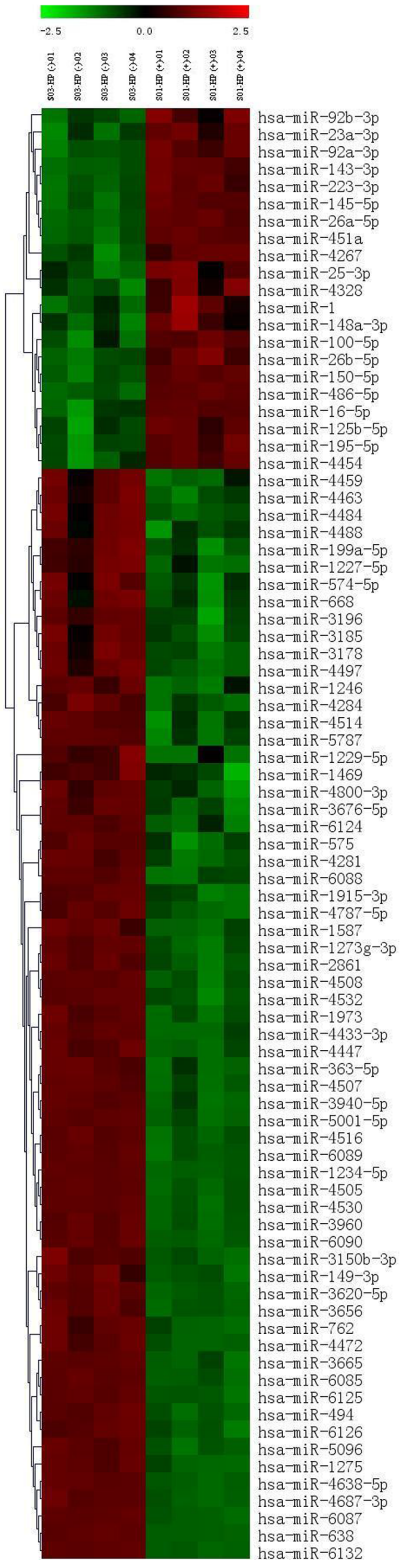
Differentially expressed miRNAs in *H. pylori*-positive and *H. pylori*-negative gastric cancer tissues

To determine the miRNA expression profiles in *H. pylori*-associated gastric cancer, we used a microarray chip to measure miRNA expression levels in 42 pairs of gastric cancer and adjacent normal tissues. Patient characteristics are given in Supplementary Table 1. To avoid bias due to sample heterogeneity, we measured gene expression for two sets of pooled samples. One set was derived from 21 *H. pylori*-positive gastric cancer tissues, and a second set was constructed from 21 *H. pylori*-negative gastric cancer tissues. We observed unique expression patterns in *H. pylori*-positive and *H. pylori*-negative gastric cancer tissues. Fifty-three miRNAs were significantly differentially expressed with a *P*<0.01. Clustering analysis revealed that *H. pylori*-positive and *H. pylori*-negative gastric cancer tissues expressed distinct patterns of miRNAs as shown in the heat map (Figure 1). When a greater than two-fold change was considered to indicate differential expression, a total of 14 miRNAs were found to be up-regulated, and 39 were found to be down-regulated, with a greater than two fold change being considered differential expression, in *H. pylori*-positive gastric cancer tissues. The miRNAs with significantly higher expression levels in *H. pylori*-positive gastric cancer tissues included miR-4328, miR-451a, miR-100-5p, miR-143-3p, and miR-145-5p (fold change>2, *P*<0.01; see Supplementary Table 2). In contrast, 37 miRNAs had significantly lower expression levels in *H. pylori*-positive gastric cancer tissues compared with *H. pylori*-negative tissues (fold change<0.5, *P*<0.01; see Supplementary Table 2).

### Expression of miR-143-3p in gastric cancer samples and cell lines

**Figure 2:**
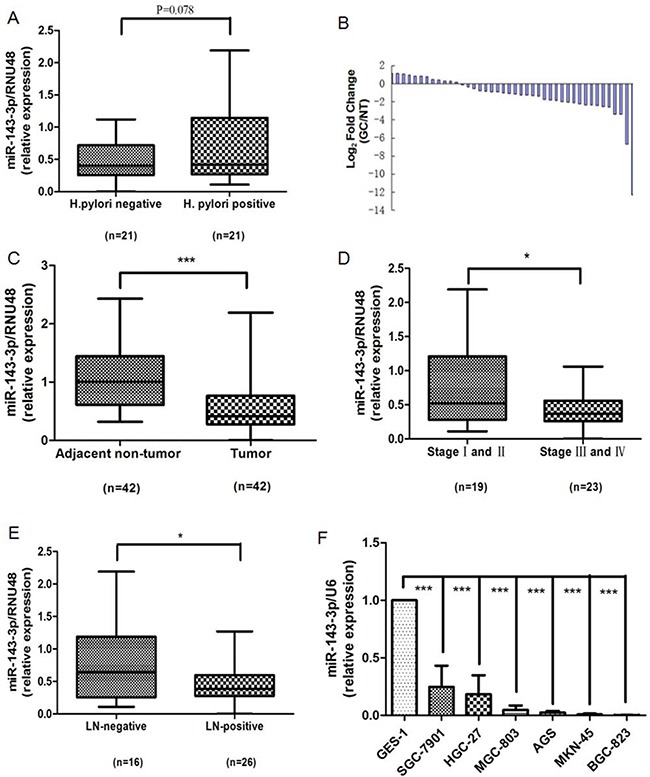
Down-regulation of miR-143-3p in gastric cancer tissues and gastric cancer cells **A**. The miR-143-3p expression level is increased in *H. pylori*-positive tumor tissues compared with the *H. pylori*-negative tumor tissues. **B**. Expression of miR-143-3p in 42 human gastric cancer (GC) samples. Data are shown as -△△CT values. **C**. Paired comparison of miR-143-3p expression level between tumor and adjacent non-tumor tissues. **D**. miR-143-3p is differentially expressed in the early stage (stages I and II) group compared with the advanced-stage (stages III and IV) group. **E**. MiR-143-3p is differentially expressed in the lymph node-negative (LN-negative) group compared with the lymph node-positive (LN-positive) group. **F**. Expression of miR-143-3p in different human gastric cancer cell lines and human gastric epithelial cell GES-1 cells. Expression of miR-143-3p was detected by qRT-PCR and was normalized against the expression of the endogenous control RNU48 (A-E) or U6 RNA (F). The horizontal lines indicate the median, 2.5% percentile and 97.5% percentile values (A, C, D, E). *P<0.05, *** *P*<0.001.

Real-time PCR analysis showed that the expression of miR-143-3p trended upward in *H. pylori*-positive gastric cancer tissues compared with *H. pylori*-negative tissues (*P*=0.078, Figure 2A), which verified the array data. We elected to pursue miR-143-3p because it was the most robustly increased miRNA in *H. pylori*-positive gastric cancer tissues. We detected the expression of miR-143-3p in 42 pairs of gastric cancer tissues and matched adjacent non-cancerous tissues, as well as in gastric cell lines. Among 42 patients with gastric cancer, approximately 73.8% (31 of 42 patients) of tumors revealed a notable reduction in the miR-143-3p levels (Figure 2B). The expression level of miR-143-3p was significantly down-regulated in gastric cancer tissues versus adjacent non-tumor tissues (*P*<0.001, Figure 2C). Next, we evaluated the association of miR-143-3p expression level with cancer stage and lymph node invasion status. We observed higher expression of miR-143-3p in early stage (stages I and II) gastric cancer tissues than in late- stage (stage III and IV) disease (*P*<0.05, Figure 2D). The expression of miR-143-3p was higher in lymph node-negative tissues relative to lymph node-positive tissues (*P*<0.05, Figure 2E). Next, we analyzed the expression levels of miR-143-3p in a normal human gastric epithelial cell line (GES-1) and in six gastric cancer cell lines (SGC-7901, MKN-45, BGC-823, MGC-803, HGC-27, and AGS). Compared with the GES-1 cell line, miR-143-3p expression was markedly reduced in the six gastric cancer cell lines (*P*<0.001, Figure 2F).

### Effects of miR-143-3p on gastric cancer cell proliferation, apoptosis, migration and invasion

**Figure 3:**
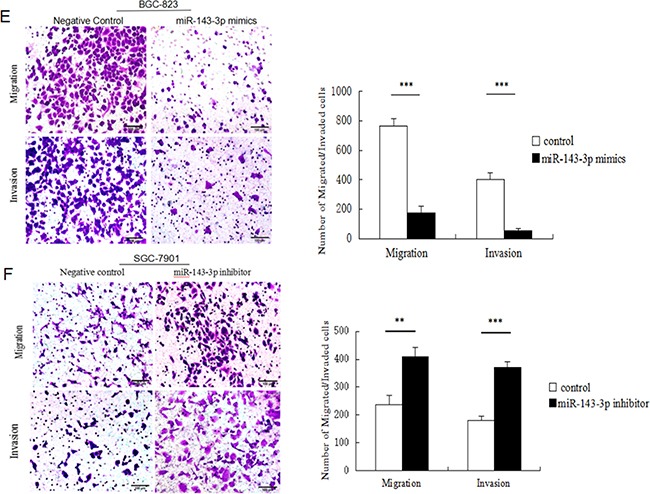
Ectopic expression of miR-143-3p in gastric cancer cells suppresses cell proliferation, induces apoptosis and inhibites cell migration and invasion **A**. MiR-143-3p expression was significantly increased by transfecting miR-143-3p mimics into BGC-823 cells and reduced by transfecting miR-143-3p inhibitor into SGC-7901 cells. As compared with their respective negative controls 48 hours after transfection and normalized to U6 snRNA. **B**. Cell viability of gastric cancer cells was examined with CCK-8 assays. Quantified relative proliferation rates are presented as a histogram in the lower panel. **C-D**. Effect of miR-143-3p on apoptosis of gastric cancer cells was examined with flow cytometry analysis. Figure is representative of 3 experiments with similar results. Late apoptosis rate, early apoptosis rate and total apoptosis rate are presented as a histogram in the lower panel. **E-F**. The transwell assay was performed to analyze the effect of miR-143-3p on the migration and invasion of BGC-823 and SGC-7901 cells. Magnification, ×200. Scale bar represents 100μm. Quantified results are presented as a histogram in the lower panel. Data represent the means±SD from 3 independent experiments. *P < 0.05, ** *P*<0.01, *** *P*<0.001.

As shown in Figure 2F, miR-143-3p expression was relatively higher in SGC-7901 cells and lower in BGC-823 cells. Therefore, we 1) overexpressed miR-143-3p in BGC-823 cells by transfecting in miR-143-3p mimics, 2) silenced miR-143-3p in SGC-7901 cells by transfecting in miR-143-3p inhibitors, and 3) performed functional assays. To assess the efficiency of the miR-143-3p transfection, we quantified the changes in miR-143-3p expression by qRT-PCR 48 hours after transfection. According to the results of qRT-PCR, the expression of miR-143-3p was significantly increased in BGC-823 cells that were transfected with miR-143-3p mimics but was decreased in SGC-7901 cells that were transfected with miR-143-3p inhibitors compared with the control miRNAs (Figure 3A).

We measured the effects of miR-143-3p expression on gastric cancer cell proliferation using CCK-8 assays (Figure 3B). In BGC-823 cells, restoration of miR-143-3p expression reduced cell viability. In contrast, miR-143-3p inhibition in SGC-7901 cells remarkably increased cell proliferation.

Apoptosis assays were performed using Annexin-V FITC/PI double-staining apoptosis detection kit. After treatment with miR-143-3p mimics, BGC-823 cells showed a significant increase in apoptosis rates (Figure 3C). Conversely, SGC-7901 cells treated with miR-143-3p inhibitors showed decreased apoptosis rates (Figure 3D).

We next examined whether miR-143-3p could affect gastric cancer cell migration and invasion by Transwell assays. The results showed that miR-143-3p re-expression distinctly abrogated the migration and invasion of BGC-823 cells (Figure 3E). Correspondingly, silencing of miR-143-3p in SGC-7901 cells promoted migration and invasion (Figure 3F).

Taken together, our data revealed that overexpression of miR-143-3p suppressed cell proliferation, induced apoptosis and inhibited cell migration and invasion. Conversely, downregulation of miR-143-3p produced opposite effects.

### AKT2 is a direct target of miR-143-3p

**Figure 4:**
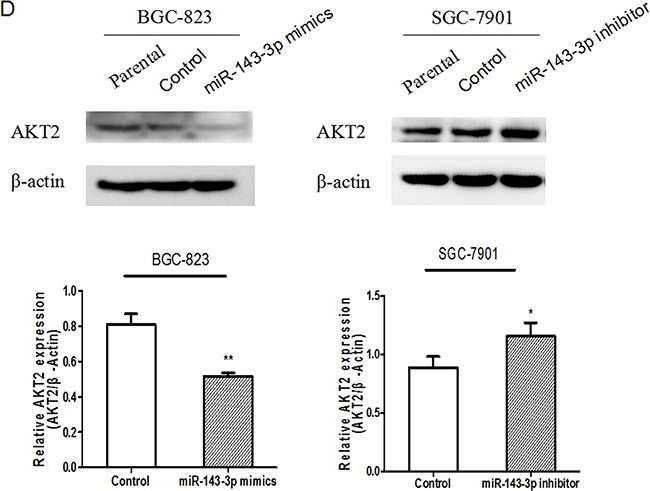
AKT2 is a direct target of miR-143-3p in gastric cancer **A**. Schematic of the construction of wild-type or mutant pGL3-AKT2 3′UTR vectors is indicated. **B**. Relative luciferase activities were analysed in HEK293T cells. Renilla luciferase vector was used as an internal control. **C**. The effect of miR-143-3p on the expression of AKT2 mRNA by qRT-PCR. β-actin served as an internal control. Data represent the mean±SD from 3 independent experiments. **D**. Related expression of AKT2 protein in cells treated with miR-143-3p mimics or its inhibitor was determined by western blot.. All *P<0.05, **P<0.01.

To search for putative protein-coding gene targets of miR-143-3p, we performed bioinformatic analyses using the miRanda, PicTar, and TargetScan algorithms. Among the candidate target genes, AKT2 was selected for further validation due to its well-described roles in proliferation and metastasis [12]. Human AKT2 contained two putative miR-143-3p target sites according to the TargetScan prediction software. To confirm the binding of miR-143-3p to the 3′-UTR of AKT2, we constructed vectors containing a wild-type or mutant 3′ UTR of AKT2 directly fused downstream of the firefly luciferase gene (Figure 4A). A dual-luciferase reporter assay revealed that miR-143-3p significantly reduced the relative luciferase activity of the wild-type AKT2 3′UTR, whereas the luciferase activity of the mutant AKT2 3′UTR remained unchanged (Figure 4B). The results demonstrated that AKT2 is a direct target of miR-143-3p.

Next, we carried out qRT-PCR and western blot analyses to explore whether the expressions of AKT2 mRNA and protein were regulated by miR-143-3p. qRT-PCR revealed that transfection of miR-143-3p mimics into BGC-823 cells resulted in a marked reduction of the expression of AKT2 mRNA, whereas transfection of the miR-143-3p inhibitor into SGC-7901 cells resulted in a marked upregulation of the expression of AKT2 mRNA (Figure 4C). Western blot analysis showed that forced expression of miR-143-3p in BGC-823 cells decreased the amount of endogenous AKT2 protein (Figure 4D). Additionally, transfection with miR-143-3p inhibitor in SGC-7901 cells, robustly increased AKT2 protein levels (Figure 4D). Moreover, AKT2 protein expression inversely correlated with miR-143-3p level in 42 pairs of gastric cancer samples using Pearson correlation analysis (r= −0.422, *P*=0.005).

### AKT2 plays a crucial role in gastric cancer cell growth, apoptosis, migration and invasion

**Figure 5:**
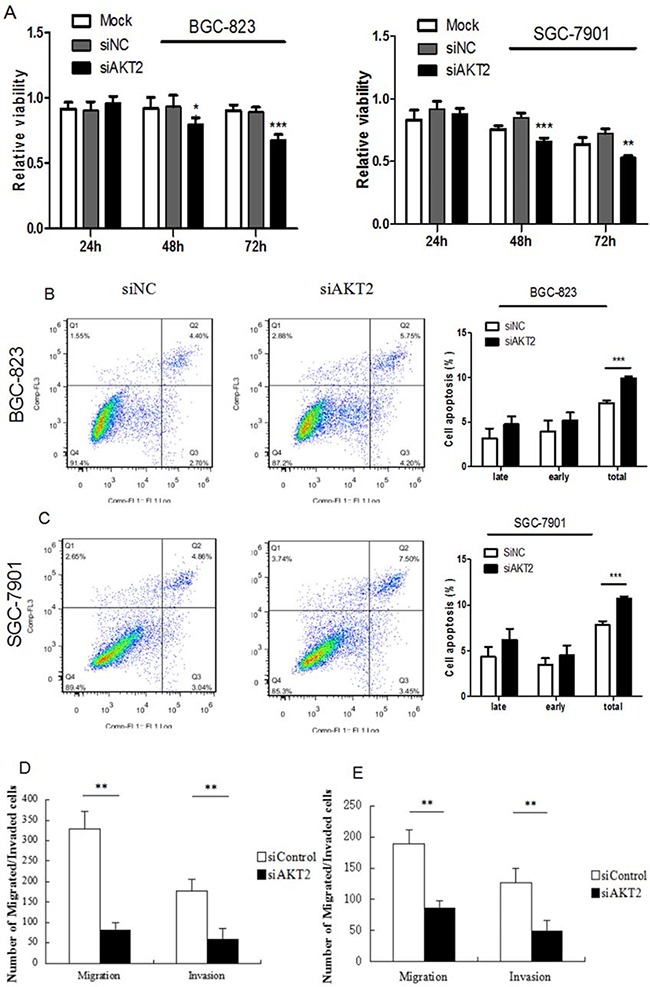
Fuctional effects of AKT2 siRNA on gastric cancer cell proliferation, apoptosis, migration and invasion **A**. Suppression of AKT2 significantly inhibited gasric cancer cell proliferation. **B-C**. Suppression of AKT2 significantly induced cell apoptosis. **D-E**. Suppression of AKT2 significantly inhibited the migration and invasion of BGC-823 and SGC-7901 cells. Data represent the means ± SD. *P < 0.05, ** *P*<0.01, *** *P*<0.001.

The effects of AKT2 on gastric cancer cells have not been characterized. To investigate the functions of AKT2 in this context, specific siRNAs against AKT2 were generated to knockdown AKT2 expression. As shown in Supplementary Figure 2, online, transfection of specific siRNAs against AKT2 into BGC-823 and SGC-7901 cells dramatically decreased AKT2 expression. CCK-8 assays showed that si-AKT2 inhibited gastric cancer proliferation (Figure 5A). Furthermore, silencing of AKT2 induced cell apoptosis and suppressed gastric cancer cell migration and invasion (Figure 5B-5E).

## DISCUSSION

Our previous study identified *H. pylori* infection as an independent favorable prognostic factor in patients with gastric cancer. In the present study, updated survival data verified those findings. minas that were differentially expressed by *H. pylori*-positive and *H. pylori*-negative gastric cancers were identified using a microarray and were confirmed by qRT-PCR. Next, we explored the expression and function of miR-143-3p, which was the most dramatically elevated miRNA in *H. pylori*-positive gastric cancer tissues. We found that miR-143-3p was diminished in gastric cancer tissues compared with adjacent non-cancerous tissues as well as in gastric cancer cell lines. We observed that miR-143-3p was down-regulated in late-stage tumors and in tumors with lymph node metastases. Furthermore, our results showed that miR-143-3p negatively regulated cell growth, apoptosis, migration and invasion. We further characterized AKT2 as a novel functional target of miR-143-3p. Together, these data might facilitate the development of novel interventional and therapeutic strategies for patients with this disease.

Matsushima et al. were the first to explore miRNA signatures in gastric mucosa with special attention paid to *H. pylori* status [11]. The aberrant expression of miRNAs in response to *H. pylori* infection has also been reported in gastric epithelial cells [13, 14]. These studies provided evidence of a nexus among miRNAs and the immune and inflammatory responses induced by *H. pylori*. To date, however, there is a paucity of data that characterizes miRNA profiles in *H. pylori*-associated gastric cancer tissues. In the current study, we identified differentially expressed miRNAs in *H. pylori*-positive and *H. pylori*-negative gastric cancer tissues. Our data indicated relatively heterogeneous gene expression profiles in *H. pylori*-positive gastric cancer compared with *H. pylori*-negative gastric cancer.

Mounting evidence has suggested that miRNAs play essential roles in the development and progression of gastric cancer. For example, miR-577 inhibits gastric cancer cell growth via the regulation of the expression of an important regulator of the E2F transcription factor 3[15]. Under hypoxic conditions, miR-18a expression was markedly downregulated in gastric carcinoma cell lines. The overexpression of miR-18a promoted apoptosis and inhibited the invasiveness of gastric cancer cells via the suppression of hypoxia-inducible factor-1α expression [16]. Nevertheless, as a known tumor-suppressive miRNA, low expression or functional confirmation of miR-143-3p in gastric cancer has been reported [17]. In the current study, we clearly demonstrated that decreased expression of miR-143-3p was significantly correlated with advanced clinical stage and tumor metastasis. This finding suggested that overexpression of miR-143-3p in gastric cancer may inhibit the invasive/metastatic phenotype. Subsequently, we confirmed that overexpression of miR-143-3p inhibited tumor growth, invasiveness and migration of BGC-823 cells (predominantly low in miR-143 expression), which was accompanied by an induction of cancer cell apoptosis. On the contrary, when miR-143-3p expression was inhibited, cell growth was enhanced and invasiveness and migration were elevated in SGC-7901 cells (with relatively high endogenous miR-143-3p expression), while apoptosis was inhibited. Taken together, these findings suggested that miR-143 regulates processes that are critical for the development of gastric cancer and that miR-143-3p exhibits a strong tumor-suppressive effect.

To the best of our knowledge, few data concerning the relationship between miR-143-3p and *H. pylori* have been published. In the current study, miR-143-3p, which exerts known tumor suppressor properties in various malignancies, was up-regulated in *H. pylori*-positive gastric cancer. In general, the overexpression of miR-143-3p can suppress tumor growth, migration, and invasion and can promote apoptosis via the targeting of AKT2. The elevated expression of the tumor suppressor miR-143-3p may lead to less aggressive biological behavior and delayed cancer progression in *H. pylori*-positive gastric cancer. *H. pylori* virulence as well as host genetic and environmental factors constitute a complex regulatory network [11, 18]. This regulatory network functions throughout the multistep process of cancer including tumorigenesis and development. Different miRNA expression patterns between *H. pylori*-positive and *H. pylori*-negative gastric cancer tissues may be determined by this complex regulatory network. The up-regulation of miR-143-3p in *H. pylori*-positive gastric cancer tissues can be directly induced by *H. pylori* infection or may be induced as a secondary effect that accompanies *H. pylori* infection. The regulation of miRNAs during *H. pylori* infection may be more complex than we imagine. Further investigation will identify which of these processes leads to miR-143 aberrant expression and will enrich our understanding of the regulation of gastric cancer occurrence and development.

The down-regulation of miR-143 has been a frequently demonstrated in various cancers. Consistent with these reports, in the present study, we also determined that miR-143-3p is down-regulated in gastric cancer tissues. Human miRNAs are frequently located at common breakpoint regions or fragile sites. Several miRNAs that are localized to deleted regions exhibit relatively decreased expression in tumor tissues [19]. Moreover, miR-143 was found to be epigenetically repressed by promoter hypermethylation [20]. In addition, several oncogenes target and negatively regulate miR-143 expression. For example, the miR-143/145 cluster was shown to be repressed by oncogenic KRAS, which is dependent on RREB1[21]. Additionally, C-MYB transactivated miR-143 by directly binding to its promoter [22]. Thus, miR-143-3p down-regulation in gastric cancer might result from multiple mechanisms, including chromosomal deletion, epigenetic regulation and transcriptional down regulation. Further investigation will identify which of these processes leads to the aberrant silencing of miR-143 and will improve our understanding of the regulation of gastric cancer metastasis.

Our data demonstrated that AKT2 is a direct downstream mediator of miR-143-3p in gastric cancer. AKT comprises a family of three serine/threonine protein kinases (Akt1, Akt2, and Akt3) which play a critical role in the regulation of a host of cellular functions. AKT2 is a pro-survival protein that is commonly overexpressed in malignancies [23, 24]. AKT2 is a downstream target of the phosphatidylinositol 3′ kinase (PI3K) pathway. The activation of the PI3K/AKT pathway is associated with aggressive cancer phenotypes and is thought to be an ideal target for the treatment of malignancies [25]. Mounting evidence indicates that AKT2 is a crucial mediator in tumorigenesis, tumor progression, metastatic spread and chemoresistance. Attoub S. and colleagues reported that AKT2 played an important role in lung cancer cell proliferation, motility, invasion and angiogenesis [12]. Pereira L et al. have characterized the implications of AKT2/Twist crosstalk on breast cancer invasiveness and chemoresistance [26]. Furthermore, AKT2 may be regulated by multiple miRNAs. The tumor-suppressor miR-29s can inhibit the invasive ability of gastric cancer cells by targeting AKT2[27]. MiR-137, which is involved in gastric cancer tumorigenesis and metastasis, may regulate AKT2-related signaling pathways [28]. To the best of our knowledge, the present study is the first to report that AKT2 is a direct target of miR-143-3p. In this study, our results demonstrated that miR-143-3p bound directly to the 3′UTR of AKT2 and decreased both the mRNA and protein expression levels of AKT2. The knockdown of AKT2 expression strongly repressed cancer cell proliferation, invasion and migration, which mimicked the effects of miR-143-3p restoration. This finding suggested the involvement of AKT2 in gastric cancer progression.

In summary, our study helped to characterize the role of miRNAs in *H. pylori*-associated gastric cancer progression. We identified an important tumor-suppressive miRNA, miR-143-3p, which was up-regulated in *H. pylori*-positive gastric cancer tissues. We observed that miR-143-3p was frequently repressed and was associated with tumor stage and lymph-node metastasis in gastric cancer. The re-expression of miR-143-3p suppressed cell growth, invasion and migration in part by directly targeting AKT2 mRNA. Further studies will help to elucidate the complicated mechanisms that underlie gastric cancer progression. The results from this work could provide insight into the use of novel therapeutic targets for the treatment of gastric cancer.

## MATERIALS AND METHODS

### Patients and specimens

Forty-two pairs of paraffin-embedded formalin fixed (FFPE) tissue samples were obtained from patients who underwent curative resection for gastric cancer between December 2007 and November 2009 at the First Affiliated Hospital of Anhui Medical University, Hefei, China. The exclusion criteria were as follows: Siewert type I adenocarcinoma of the cardia, distant metastasis, received neoadjuvant treatment before surgery, underwent non-resective surgery, and used medications effective against *H. pylori*. This study was approved by the Institutional Human Subject Research Review Committee of Anhui Medical University (Anhui, China). Written informed consent was obtained from all of the participants.

Tumor and non-neoplastic tissues were collected from the resected stomach specimens in the operating room within 30 minutes of the removal of the stomach. Non-neoplastic tissue was taken from the antrum and corpus mucosa at a distance of at least 5 cm from the tumor, which was stripped along the side of the lesser curvature. If the neoplasm involved the entire antrum, we selected the non-neoplastic mucosa from the middle or upper third of the stomach. All harvested stomach specimens were immediately fixed in 10% neutral-buffered formalin, embedded in paraffin, and cut into 4-μm-thick sections for hematoxylin-eosin (H-E) and immunohistochemical (IHC) staining.

IHC staining was used for the detection of *H. pylori*[29–31]. *H. pylori* infection status was graded according to the visual analog scale of the Updated Sydney System and was categorized as either negative (normal) or positive (mild, moderate, marked) [32]. Only when the test of both the tumor and non-neoplastic samples was negative, were the patients regarded as negative for *H. pylori*; otherwise, they were regarded as positive for *H. pylor*i. The methods for *H. pylori* detection were described in detail in our previous study [3].

### Cell culture and transfection

The non-malignant gastric epithelial cell line GES-1, and the human gastric cancer cell lines AGS, MGC-803, BGC-823, SGC-7901, HGC-27, MKN-45, and HEK-293T were purchased from the Cell Resource Centre, Shanghai Institute of Biochemistry and Cell Biology at the Chinese Academy of Sciences. The cells were cultured by standard methods in RPMI-1640/F12 medium (Invitrogen, California, USA) supplemented with 10% fetal bovine serum, in a 37°C humidified chamber with 5% CO_2_. MiR-143-3p mimics and inhibitors used in this study were purchased from Ambion. For gain-of-function experiments, BGC-823 cells were transfected with 50 nM miR-143-3p mimics (Cat #4464066, ID MC10883, Applied Biosystems, California, USA) or with miRNA mimic negative controls (Cat #4464058). For loss-of-function experiments, SGC-7901 cells were transfected with 50 nM of miR-143-3p inhibitors (Cat #4464084, ID: MH10883, Applied Biosystems, California, USA) or with miRNA inhibitor negative controls (Cat #4464076) in parallel. The knockdown of AKT2 was performed using AKT2 small interfering RNA (siRNA) (100 nM, GenePharma). The cells were plated in 6-well or 96-well plates, and all cells were transfected using Lipofectamine 3000 transfection reagent (Invitrogen, California, USA) according to the manufacturer's instructions. Transfected cells were used in functional assays or for RNA/protein extraction, as specified.

### RNA extraction and μParaflo microRNA microarray assay

Total RNA was extracted from FFPE tissue samples using a RecoverAll® kit (Ambion; RecoverAll® Total Nucleic Acid Isolation Kit for FFPE; cat no. AM1975) according to the manufacturer's protocol. Total RNA was extracted from cells using TRIzol reagent (Invitrogen, California, USA).

Forty-two gastric cancer tissues and corresponding adjacent normal tissues were selected for miRNA microarray analysis. The microarray was custom-built by LC Sciences (Houston, TX) using Sanger miRBase version 19.0, which covered 2019 unique mature human miRNA probes (http://www.sanger.ac.uk/Software/Rfam/mirna/). The biological repeat number was the same as the sample number, and each sample had four technical replicates. A 5 μg total RNA sample was extracted as described above. Hybridization was performed overnight on a μParaflo^®^ microfluidic chip using a micro-circulation pump (Atactic Technologies) [33]. After RNA hybridization, tag-conjugating Cy3 dye was circulated through the microfluidic chip for dye staining. Fluorescent images were collected using a laser scanner (GenePix 4000B, Molecular Devices) and digitized using Array-Pro image analysis software (Media Cybernetics). The output intensity data were analyzed by first subtracting the background and then normalizing the signals using a LOWESS filter (locally weighted regression) [34]. The data were analyzed by Student's t-test to identify differentially expressed miRNAs. Differences in miRNA expression between two groups were considered significant if the fold change of expression values was >2.0 and if the *P* value was <0.05 according to the *t* test.

### Quantitative real-time PCR analysis (qRT-PCR)

MiR-143-3p and AKT2 mRNA expression levels were determined by qRT-PCR using the 7900HT Real-Time PCR System (Applied Biosystems, CA, USA). Real-time quantitative RT-PCR (qRT-PCR) analysis was performed to detect miRNA or mRNA expression. In all, 1 μg of total RNA was reverse transcribed to cDNA using a RevertAid Premium First Strand cDNA Synthesis Kit (Fermentas, Burlington, Canada). The cDNA was amplified by real-time PCR with a SYBR Green PCR master mix kit (Invitrogen, California, USA). RNU48 snRNA, U6 snRNA or β-actin was used as an endogenous control. The primers used to detect miRNA or mRNA are listed in Supplementary Table 3, which is available online. Gene expression was measured in triplicate, and the data were processed using the 2^−△△Ct^ method and normalized to the controls.

### Protein extraction and western blot analysis

The cells were lysed using RIPA buffer supplemented with protease inhibitors. For electrophoresis, equal amounts of cell lysate were loaded. The proteins were subjected to SDS-PAGE and transferred onto polyvinylidene difluoride (PVDF) membranes (Bio-Rad Laboratories), which were blocked and probed with anti-AKT2 antibodies (ab66129, Abcam) followed by an HRP-conjugated secondary antibody. The proteins of interest were visualized using ECL substrate. β-actin served as the loading control.

### CCK-8 cell proliferation assay

Cell proliferation rates were measured by Cell Counting Kit- 8 (CCK-8). Briefly, 3 × 10^3^ cells were seeded into each well of a 96-well plate and transfected. At different time points (24, 48 and 72 hours post-transfection), 10 μl of CCK-8 reagent was added to each well. The plates were incubated at 37˚C for 1 hour. Absorbance at 450 nm was determined in each well by a microplate reader. At least three independent experiments were performed and each experimental group consisted of six replicate wells for each time point.

### Transwell cell migration and invasion assays

For cell migration, Transwell inserts with 8.0 μm pores (Corning, NY, USA) were placed in a 24-well plate with 750 μl of culture medium and 10% FBS in the lower chamber as a chemoattractant. The cells were harvested 48 hours after transfection. Next, 1 × 10^5^ cells resuspended in 100 μl serum-free medium were added to the upper chamber. The cells were then incubated for 24 hours (SGC-7901 cells) or 48 hours (BGC-823 cells) at 37°C with 5% CO_2_ to measure the effects of miR-143-3p mimics or inhibitors on cell migration potential.

For cell invasion, the inserts were pre-coated with 60 μl of freshly diluted extracellular Matrigel (cat. no. 356234, BD Biosciences; 1:7 dilution in serum-free medium), placed in a 24-well plate, and incubated at 37°C for three hours. The cells were incubated for 72 hours (SGC-7901 and BGC-823 cells) to measure the effect of miR-143-3p mimics or inhibitors on cell invasion potential. Cotton swabs were used to remove the cells or matrix on the top surface of the membrane. Migrated or invaded cells were fixed in 90% ethanol and stained with 0.1% crystal violet. Five low-magnification areas (×100) were randomly selected and examined to determine the cell numbers.

### Apoptosis assays

To detect apoptotic cells via flow cytometry (Cytomics FC 500; Beckman Coulter, CA 92821, USA), an Annexin-V fluorescein isothiocyanate/propidium iodide ((FITC/PI) double-staining apoptosis detection kit was used, according to the manufacturer's protocols. Apoptosis assays were performed using an Annexin-V FITC apoptosis kit. Briefly, cells were collected 48 hours after transfection, washed with PBS twice, and re-suspended in 400 μl 1×Binding Buffer at 1 × 10^6^ cells/ml. Then, 5 μl of Annexin-V FITC was added to the cell suspensions, gently mixed, and incubated for 15 minutes in the dark followed by the addition of 5 μl PI. After 5 minutes of incubation at room temperature, the samples were analyzed by flow cytometry and quantified by FlowJo software version 7.6.2 (http://www.flowjo.com/index.php). At least three independent experiments were performed in triplicate.

### Luciferase assay

All the reporter vectors were constructed using the pmirGLO Dual-Luciferase miRNA Target Expression Vector (Promega Corporation, Madison, WI). The wild-type and mutated putative miR-143-3p binding sites of AKT2 were cloned to the 3′- UTR downstream of the luc2 firefly luciferase gene according to the manufacturer's instructions.

HEK293T cells were co-transfected with the pmirGLO reporter plasmids (15 ng) and miR-143-3p mimics or miR-143-3p NC (120pmol) using Lipofectamine 2000 Transfection Reagent (Invitrogen). The cells were collected 24 and 48 hours after transfection and the firefly and Renilla luciferase luminescence signals were determined using a Dual-GLO Luciferase Assay System (Promega Corporation). Data were analyzed by dividing firefly luciferase activity by Renilla luciferase activity for each transfectant and were further normalized to the empty pmirGLO vector transfectant.

### Statistical analyses

All results were confirmed in at least three independent experiments, but data from only one representative experiment are shown. The statistical analyses were performed using the SPSS 13.0 software. Differences between two groups were compared using Pearson's chi-square test for qualitative variables. For continuous variables, differences among groups were estimated by Student's *t* test, one-way ANOVA or non-parametric test. A Pearson correlation analysis was used to establish the relationship between tumor miR-143-3p expression and AKT2 expression levels. *P* values < 0.05 were considered to be statistically significant.

## SUPPLEMENTARY MATERIALS FIGURES AND TABLES





## References

[1] Forman D (1991). The etiology of gastric cancer. IARC Sci Publ.

[2] Meimarakis G, Winter H, Assmann I, Kopp R, Lehn N, Kist M, Stolte M, Jauch KW, Hatz RA (2006). Helicobacter pylori as a prognostic indicator after curative resection of gastric carcinoma: a prospective study. Lancet Oncol.

[3] Wang F, Sun GP, Zou YF, Zhong F, Ma T, Li XQ, Wu D (2013). Helicobacter pylori infection predicts favorable outcome in patients with gastric cancer. Curr Oncol.

[4] Wang F, Sun G, Zou Y, Zhong F, Ma T, Li X (2013). Protective role of Helicobacter pylori infection in prognosis of gastric cancer: evidence from 2,454 patients with gastric cancer. PLoS One.

[5] He L, Hannon GJ (2004). MicroRNAs: small RNAs with a big role in gene regulation. Nat Rev Genet.

[6] Yang Q, Jie Z, Cao H, Greenlee AR, Yang C, Zou F, Jiang Y (2011). Low-level expression of let-7a in gastric cancer and its involvement in tumorigenesis by targeting RAB40C. Carcinogenesis.

[7] Zhu ED, Li N, Li BS, Li W, Zhang WJ, Mao XH, Guo G, Zou QM, Xiao B (2014). miR-30b, down-regulated in gastric cancer, promotes apoptosis and suppresses tumor growth by targeting plasminogen activator inhibitor-1. PLoS One.

[8] Yu J, Feng J, Zhi X, Tang J, Li Z, Xu Y, Yang L, Hu Z, Xu Z (2015). Let-7b inhibits cell proliferation, migration, and invasion through targeting Cthrc1 in gastric cancer. Tumour Biol.

[9] Guo J, Miao Y, Xiao B, Huan R, Jiang Z, Meng D, Wang Y (2009). Differential expression of microRNA species in human gastric cancer versus non-tumorous tissues. J Gastroenterol Hepatol.

[10] Li X, Luo F, Li Q, Xu M, Feng D, Zhang G, Wu W (2011). Identification of new aberrantly expressed miRNAs in intestinal-type gastric cancer and its clinical significance. Oncol Rep.

[11] Matsushima K, Isomoto H, Inoue N, Nakayama T, Hayashi T, Nakayama M, Nakao K, Hirayama T, Kohno S (2011). MicroRNA signatures in Helicobacter pylori-infected gastric mucosa. Int J Cancer.

[12] Attoub S, Arafat K, Hammadi NK, Mester J, Gaben AM (2015). Akt2 knock-down reveals its contribution to human lung cancer cell proliferation, growth, motility, invasion and endothelial cell tube formation. Sci Rep.

[13] Liu Z, Xiao B, Tang B, Li B, Li N, Zhu E, Guo G, Gu J, Zhuang Y, Liu X, Ding H, Zhao X, Guo H (2010). Up-regulated microRNA-146a negatively modulate Helicobacter pylori-induced inflammatory response in human gastric epithelial cells. Microbes Infect.

[14] Zhou X, Xu G, Yin C, Jin W, Zhang G (2014). Down-regulation of miR-203 induced by Helicobacter pylori infection promotes the proliferation and invasion of gastric cancer by targeting CASK. Oncotarget.

[15] Yu Z, Zhang W, Deng F (2015). MicroRNA-577 inhibits gastric cancer growth by targeting E2F transcription factor 3. Oncol Lett.

[16] Wu F, Huang W, Wang X (2015). microRNA-18a regulates gastric carcinoma cell apoptosis and invasion by suppressing hypoxia-inducible factor-1α expression. Exp Ther Med.

[17] Wu XL, Cheng B, Li PY, Huang HJ, Zhao Q, Dan ZL, Tian DA, Zhang P (2013). MicroRNA-143 suppresses gastric cancer cell growth and induces apoptosis by targeting COX-2. World J Gastroenterol.

[18] Blaser MJ, Atherton JC (2004). Helicobacter pylori persistence: biology and disease. J Clin Invest.

[19] Calin GA, Sevignani C, Dumitru CD, Hyslop T, Noch E, Yendamuri S, Shimizu M, Rattan S, Bullrich F, Negrini M, Croce CM (2004). Human microRNA genes are frequently located at fragile sites and genomic regions involved in cancers. Proc Natl Acad Sci USA.

[20] Dou L, Zheng D, Li J, Li Y, Gao L, Wang L, Yu L (2012). Methylation-mediated repression of microRNA-143 enhances MLL-AF4 oncogene expression. Oncogene.

[21] Kent OA, Fox-Talbot K, Halushka MK (2013). RREB1 repressed miR-143/145 modulates KRAS signaling through downregulation of multiple targets. Oncogene.

[22] Wang W, Wu S, Shi Y, Miao Y, Luo X, Ji M, Yao K, He J (2015). c-MYB regulates cell growth and DNA damage repair through modulating MiR-143. FEBS Lett.

[23] Rychahou PG, Kang J, Gulhati P, Doan HQ, Chen LA, Xiao SY, Chung DH, Evers BM (2008). Akt2 overexpression plays a critical role in the establishment of colorectal cancer metastasis. Proc Natl Acad Sci USA.

[24] Archewa P, Pata S, Chotjumlong P, Supanchart C, Krisanaprakornkit S, Iamaroon A (2015). Akt2 and p-Akt overexpression in oral cancer cells is due to a reduced rate of protein degradation. J Investig Clin Dent.

[25] Manning BD, Cantley LC (2007). AKT/PKB signaling: navigating downstream. Cell.

[26] Pereira L, Horta S, Mateus R, Videira MA (2015). Implications of Akt2/Twist crosstalk on breast cancer metastatic outcome. Drug Discov Today.

[27] Zhang H, Cheng Y, Jia C, Yu S, Xiao Y, Chen J (2015). MicroRNA-29s could target AKT2 to inhibit gastric cancer cells invasion ability. Med Oncol.

[28] Wu L, Chen J, Ding C, Wei S, Zhu Y, Yang W, Zhang X, Wei X, Han D (2015). MicroRNA-137 Contributes to Dampened Tumorigenesis in Human Gastric Cancer by Targeting AKT2. PLoS One.

[29] Lash RH, Genta RM (2016). Routine Anti-Helicobacter Immunohistochemical Staining is Significantly Superior to Reflex Staining Protocols for the Detection of Helicobacter in Gastric Biopsy Specimens. Helicobacter.

[30] Jonkers D, Stobberingh E, de Bruine A, Arends JW, Stockbrügger R (1997). Evaluation of immunohistochemistry for the detection of Helicobacter pylori in gastric mucosal biopsies. J Infect.

[31] Batts KP, Ketover S, Kakar S, Krasinskas AM, Mitchell KA, Wilcox R, Westerhoff M, Rank J, Gibson J, Mattia AR, Cummings OW, Davison JM, Naini BV, Rodger C (2013). Haggitt Gastrointestinal Pathology Society, and Haggitt Gastrointestinal Pathology Society. Appropriate use of special stains for identifying Helicobacter pylori: Recommendations from the Rodger C. Am J Surg Pathol.

[32] Dixon MF, Genta RM, Yardley JH, Correa P (1996). Classification and grading of gastritis. The updated Sydney System International Workshop on the Histopathology of Gastritis, Houston 1994.

[33] Zhu Q, Hong A, Sheng N, Zhang X, Matejko A, Jun KY, Srivannavit O, Gulari E, Gao X, Zhou X (2007). microParaflo biochip for nucleic acid and protein analysis. Methods Mol Biol.

[34] Bolstad BM, Irizarry RA, Astrand M, Speed TP (2003). A comparison of normalization methods for high density oligonucleotide array data based on variance and bias. Bioinformatics.

