# Supplementary material for: MicroRNA-143-3p, up-regulated in H. pylori-positive gastric cancer, suppresses tumor growth, migration and invasion by directly targeting AKT2
Source: Oncotarget. 2017 Feb 23;8(17):28711–24. doi: 10.18632/oncotarget.15646 (PMC5438685; doi:10.18632/oncotarget.15646)
Supplement: Supplementary file 1 [file oncotarget-08-28711-s001.pdf]

MicroRNA-143-3p, up-regulated in *H. pylori*-positive gastric cancer, suppresses tumor growth, migration and invasion by directly targeting AKT2

SUPPLEMENTARY FIGURES AND TABLES

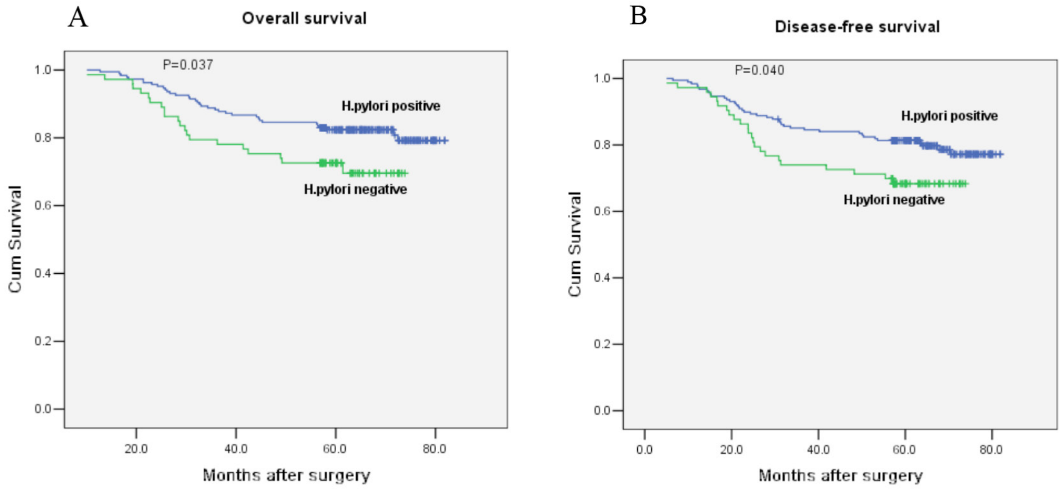

Supplementary Figure 1: Kaplan-Meier analysis of Overall survival A. and Disease-free survival B. according to *H. pylori* status.

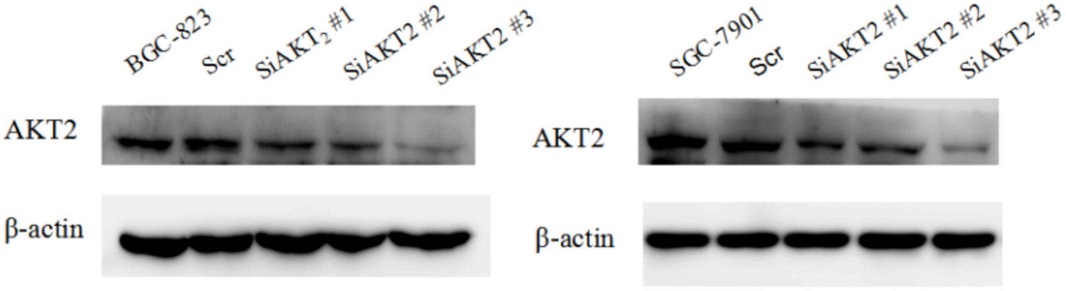

Supplementary Figure 2: Knockdown of AKT2 by siRNA was confirmed by western blot in BGC-823 and SGC-7901 cells. β-actin served as an internal control.

Supplementary Table 1: Forty-two Patients' clinicopathological characteristics

Characteristic	*H. pylori*		
	**Positive (%)**	**Negative (%)**	***P* value**
Age (years) 58 (29-76)			0.747
<58	8 (38.1)	7 (33.3)	
≥58	13 (61.9)	14 (66.7)	
Sex			0.292
Male	14 (66.7)	17 (81.0)	
Female	7 (33.3)	4 (19.0)	
Tumor location			0.006
cardia	1 (4.8)	4 (19.0)	
fundus	0 (0.0)	4 (19.0)	
corpus	9 (42.9)	10 (47.6)	
antrum	11 (52.4)	3 (14.3)	
Grade			0.513
moderate	6 (28.6)	8 (38.1)	
poor	15 (71.4)	13 (61.9)	
Lauren classification			0.946
intestinal	12 (57.1)	11 (52.4)	
diffuse	7 (33.3)	8 (38.1)	
mixed	2 (9.5)	2 (9.5)	
TNM status			
T1	4 (19.0)	0 (0.0)	0.024
T2	5 (23.8)	3 (14.3)	
T3	12 (57.1)	16 (76.2)	
T4	0 (0.0)	2 (9.5)	
N0	10 (47.6)	6 (28.6)	0.440
N1	9 (42.9)	12 (57.1)	
N2	2 (9.5)	3 (14.3)	
M0	21 (100.0)	21 (100.0)	
M1	0 (0.0)	0 (0.0)	
UICC stage			
I	6 (28.6)	1 (4.8)	0.041
II	7 (33.3)	5 (23.8)	
III	8 (38.1)	13 (61.9)	
IV	0 (0.0)	2 (9.5)	
Mean OS (months)	69.9(95%CI: 61.3-78.4)	31.1(95%CI:24.9-37.2)	<0.01

Abbreviations: TNM, TNM staging; OS, overall survival.

Supplementary Table 2: Differential expressed microRNAs in *H. pylori*-positive gastric cancer tissues compared with *H. pylori*-negative gastric cancer tissues.

See Supplementary File 1

Supplementary Table 3: PCR primers

Gene	Sequences
miR-143-3p	Forward:5'GCGGCGGTGAGATGAAGC3'
	Reversed:5'CAGTGCAGGGTCCGAGGTAT3'
RNU48	Forward:5'CCCCAGGTAACTCTGAGTGTGTC3'
	Reversed:5'CGCAGGGTCCGAGGTATTC3'
AKT2	Forward:5' CAAGGATGAAGTCGCTCACACAGT 3'
	Reversed:5' GGCATACTCCATCACAAAGCACA 3'
β-actin	Forward:5'GGGAAATCGTGCGTGACATTAAGG3'
	Reversed:5'CAGGAAGGAAGGCTGGAAGAGTG3'
