# Supplementary material for: MicroRNA-143-3p, up-regulated in H. pylori-positive gastric cancer, suppresses tumor growth, migration and invasion by directly targeting AKT2
Source: Oncotarget. 2017 Feb 23;8(17):28711–24. doi: 10.18632/oncotarget.15646 (PMC5438685; doi:10.18632/oncotarget.15646)
Supplement: Supplementary file 2 [file oncotarget-08-28711-s002.docx]

| **Supplementary Table 2**. Differential expressed microRNAs in *H. pylori*-positive gastric cancer tissues compared with *H. pylori*-negative gastric cancer tissues | | |
| --- | --- | --- |
| microRNA | Fold change^*^ | *P*-value |
| hsa-miR-4328 | 3.91 | 3.96E-03 |
| hsa-miR-451a | 2.61 | 3.85E-04 |
| hsa-miR-100-5p | 2.22 | 6.54E-03 |
| hsa-miR-143-3p | 2.17 | 2.00E-05 |
| hsa-miR-145-5p | 2.05 | 1.93E-04 |
| hsa-miR-1273g-3p | 0.48 | 7.88E-04 |
| hsa-miR-4787-5p | 0.47 | 2.70E-05 |
| hsa-miR-3150b-3p | 0.42 | 4.48E-05 |
| hsa-miR-4497 | 0.42 | 9.18E-04 |
| hsa-miR-3178 | 0.39 | 2.54E-03 |
| hsa-miR-3960 | 0.39 | 4.73E-06 |
| hsa-miR-638 | 0.37 | 1.43E-08 |
| hsa-miR-6087 | 0.37 | 4.24E-05 |
| hsa-miR-1246 | 0.33 | 2.64E-03 |
| hsa-miR-4516 | 0.32 | 5.23E-05 |
| hsa-miR-3665 | 0.32 | 4.07E-04 |
| hsa-miR-6090 | 0.32 | 7.19E-07 |
| hsa-miR-4508 | 0.30 | 5.93E-04 |
| hsa-miR-3656 | 0.30 | 6.82E-06 |
| hsa-miR-3196 | 0.30 | 2.98E-03 |
| hsa-miR-1587 | 0.28 | 6.64E-05 |
| hsa-miR-4488 | 0.27 | 7.48E-03 |
| hsa-miR-6089 | 0.26 | 2.77E-04 |
| hsa-miR-6085 | 0.26 | 1.63E-04 |
| hsa-miR-4507 | 0.24 | 5.70E-04 |
| hsa-miR-3620-5p | 0.23 | 1.00E-06 |
| hsa-miR-5096 | 0.23 | 7.52E-06 |
| hsa-miR-4505 | 0.23 | 1.12E-04 |
| hsa-miR-4530 | 0.22 | 1.47E-04 |
| hsa-miR-3940-5p | 0.21 | 1.00E-03 |
| hsa-miR-1234-5p | 0.21 | 1.80E-06 |
| hsa-miR-6125 | 0.20 | 1.88E-04 |
| hsa-miR-5001-5p | 0.18 | 3.04E-04 |
| hsa-miR-4447 | 0.17 | 2.42E-05 |
| hsa-miR-494 | 0.16 | 6.57E-06 |
| hsa-miR-4484 | 0.14 | 9.25E-03 |
| hsa-miR-5787 | 0.12 | 2.91E-03 |
| hsa-miR-4687-3p | 0.12 | 1.88E-06 |
| hsa-miR-1275 | 0.11 | 6.12E-05 |
| hsa-miR-4532 | 0.11 | 1.04E-03 |
| hsa-miR-4472 | 0.09 | 6.38E-05 |
| hsa-miR-4638-5p | 0.06 | 1.99E-07 |
| hsa-miR-4514 | 0.05 | 5.84E-03 |
| ^*^Fold change: *H pylori* positive versus *H.pylori* negative.. | | |
